# Emotional intelligence and self-esteem among Saudi Arabian and Indian nursing students: findings from two countries

**DOI:** 10.1186/s12912-024-02022-8

**Published:** 2024-05-24

**Authors:** Lizy Sonia Benjamin, Eddieson Pasay an, K Vijayalakshmi, Aida Sanad Alqarni, Abeer Aseeri, Amal Alsulami, Ferdinand Gonzales, Romeo Mostoles, Richard Maestrado, Benito Areola, Analita Gonzales, Sumathi Robert Shanmugam

**Affiliations:** 1 College of Nursing, King Khalid University, Abha City, Saudi Arabia; 2 Research Development, Apollo College of Nursing, The Tamilnadu Dr MGR Medical University, Chennai City, India; 3 College of Nursing, Muhyil Asir Branch, King Khalid University, Abha City, Saudi Arabia; 4 Department of Community and Mental Health Nursing Sciences, College of Nursing, Taif University, Taif City, Saudi Arabia; 5 College of Nursing, University of Hail, Hail City, Saudi Arabia; 6 Faculty, College of Nursing, Shaqra University, Riyadh City, Kingdom of Saudi Arabia; 7 Faculty of Nursing, University of Tabuk, Tabuk City, Saudi Arabia; 8 Department of Maternity and Pediatric Nursing, Princess Nourah Bint Abdulrahman University, Riyadh City, Saudi Arabia; 9 Department of Nursing Administration, King Khalid University, Abha City, Saudi Arabia

**Keywords:** Emotional intelligence, Self-esteem, Nursing students, Demographics, Saudi Arabia, India

## Abstract

**Introduction:**

Understanding the emotional intelligence (EI) and self-esteem of Indian and Saudi nursing students is important because their future nurse–patient relationships may be influenced by factors related to their unique cultural contexts. Hence, this study sought to investigate the EI and self-esteem of nursing students in Saudi Arabia and India.

**Methods:**

A descriptive–comparative research design was used to compare 660 nursing students from Saudi Arabia and India who were enrolled in the study from September 2022 to January 2023. Nursing students were recruited from University A in Riyadh and University B in Abha, Saudi Arabia, and from University C in India.

**Results:**

The study found significant differences in EI scores based on year of study (*p* < .011), age (*p* < .024), residence (*p* < .005), and academic performance (*p* < .008). Students in later years, over 20 years old, from urban areas, and with good grades, had higher EI scores. Conversely, only age showed a significant difference in the self-esteem scores (*p* < .002). The year of study (*p* > .670), residence (*p* > .430), and academic performance (*p* > .526) did not significantly affect self-esteem. Finally, urban residence and good academic performance were significant predictors of EI (*p* < .005), while none of the demographics predicted self-esteem (*p* > .005).

**Conclusion:**

Higher emotional intelligence among nursing students can be associated with several variables, such as being in the higher years of study, older age, residing in an urban area, and good academic performance, whereas self-esteem appears to be hardly affected by these indicators but probably influenced by other aspects that were not measured. This implies that educators in relation to self-esteem should know the relationship between emotional intelligence and nursing practice besides healthcare establishments; they need to enhance their teaching methods so that learners can have more resilient attitudes towards work, provide quality patient care, and promote a better learning environment for nurses who will become stronger professionally in the future.

## Introduction

Emotional intelligence (EI) is an individual’s ability to understand, use, and control emotions through effective communication with others [1]. Thus, for nursing students, self-awareness is essential, as it helps them manage their feelings better and show empathy while learning the practical skills needed throughout their studies and preparing for future practice. Self-esteem refers to what a person thinks about themselves; it can be described as an evaluation based on emotions that determines how much people value themselves positively or negatively [2]. In this regard, among student nurses undertaking training programs in higher education institutions, attributes such as emotional intelligence and self-esteem should not only foster success, but also enhance competence levels among learners, which eventually leads to holistic development within individuals [3]. Nurses’ EI has been found to affect performance in various areas, including patient satisfaction, teamwork, and conflict resolution [4]. Hence, there is a need to understand the connections between different aspects of psychological well-being among trainee nurses so that appropriate interventions can be implemented if necessary.

To better prepare nurses, nursing programs should provide students with the necessary resources to succeed in school, gain clinical skills, and handle emotional strain at work by enhancing their EI and self-esteem. Previous studies have indicated that emotional intelligence (EI) and self-esteem among nursing students significantly affect their academic performance, clinical competence, and general health status. For example, EI enables patients to effectively control their emotions while dealing with patients, leading to improved clinical outcomes [5, 6]. Therefore, those with higher EI levels are better placed to manage the emotional demands of this profession. However, according to Budler [7], as one progresses in their education level, the more likely they are to have an increased level of emotional intelligence, implying that these things can be taught or enhanced over time. Moreover, there is a positive correlation between self-esteem and emotional intelligence among nursing students, thus showing that individuals with higher levels of confidence tend towards have good mental health even when faced with stressors such as exams [8]. Similarly, those who possess high EIs together with strong self-esteem tend not only to report themselves being less stressed but also experiencing lower rates of depression anxiety, thus promoting wellness within oneself while studying hard for challenging nursing courses. Empathy, which falls under the components of EIs, is crucially important since it helps healthcare workers understand their clients’ needs better, providing compassionate care for them. Nevertheless, previous research argues that during training period nurses may lose touch with empathy due lack thereof which implies need for developing other-related competences among trainee nurses so as not kill such emotions entirely in them [9].

The largest group of nurses in Saudi Arabia is foreign, and the majority are from India. In fact, More than half (60–70%) of all working nurses in the country come from abroad [10]. This is reflected in the policy: Saudi Arabia actively recruits staff from countries such as India and the Philippines [4]. Various studies have been conducted on this topic. They look into different aspects, such as culture shock, language barriers, and professional adaptation, regarding these groups’ experiences while working there [11]. The high number of Indian nurses found within Saudi Arabia is due to attempts made by them to meet their increasing demand for healthcare workers, which could be seen as an example where one country needs another’s help because it lacks enough staff members. More specifically, it has been estimated that by 2025, Saudi Arabia will need an additional 1,000,000 nurses to bridge the current gap [10].

Given that both countries have distinct cultures, emotional intelligence and self-esteem among Indian nurses also hold true among those who work in Saudi Arabia. However, to the best of our knowledge, no studies have investigated how these two variables are related, especially within the context of KSA [4]. Such a study should generate multiple outcomes among nursing professionals based on an understanding of particular settings characterized by different manifestations. For example, it is important to foster emotional intelligence and self-esteem among Indian Nurses working in Saudi Arabia to achieve success with nursing education programs as well as integration into the Saudi Arabian health care system. Demographic factors affecting cognitive development differ significantly between India and Saudi Arabia, which calls for need-based interventions that cater to all students undertaking nursing courses in either country or both nations. Recognizing this difference, therefore, provides a foundation towards developing strategies aimed at supporting every student in nursing courses in Saudi Arabia or India. Prior reviews suggest that student nurses have average emotional intelligence and a neutral tendency toward critical thinking; hence, this provides empirical evidence supporting the requirement for Emotional Intelligence among future frontline healthcare providers [12].

Previously, it was found that there is a significant relationship between EI and self-esteem among university students in Pakistan [13], which is similar to what was realized with student nurses participating in a cross-sectional study in Saudi Arabia [14] aimed at determining their levels of emotional intelligence (EI) as well as self-esteem, and both were established to be high. Therefore, this finding demonstrates how complex an emotional intelligence construct can be within nursing students of middle-eastern universities, particularly when viewed against the backdrop of a specific cultural milieu such as the Saudi Arabian context. It is worth noting that culturally sensitive assessment tools should be used for measuring EI [15, 16], which becomes even more crucial in these countries. Therefore, there is a need for further inquiry into areas where current knowledge about them lacks completion, especially for Indian and Saudi Arabian undergraduate nurses. An integration of best practices within nursing education programs requires a comprehensive understanding of cultures between these two countries towards developmentally enhancing Emotionally Intelligent caregiving skills while fostering self-esteem among staff caring for patients from different backgrounds, but also increasing the number and diversity of future nurses in Saudi Arabia.

Although this study only involved undergraduate students, we understand that our results cannot be applied to all nursing students. However, it is important to know how emotional intelligence (EI) and self-esteem interact at the start of nursing school, since this foundational period shapes a person’s professional identity and overall health. We also realize that more work needs to be done in order for us to comprehend what happens with these dynamics as they progress through various levels of education. When choosing India and Saudi Arabia as our focus countries, we were motivated by their unique cultural settings and rapidly expanding numbers of nursing institutions along with different healthcare systems. By doing so, the researchers were able to see if there were any cultural elements or educational frameworks that may impact the correlation between EI and self-esteem among nursing scholars. This study aims to fill this void while providing insights into culturally appropriate educational programs for fostering EI in addition to self-worth among nurses from both India and Saudi Arabia. Moreover, appreciating these dynamics in both nations becomes even more relevant because some Indian nurses might find themselves working in Saudi Arabian healthcare establishments. This investigation can also help develop strategies on how best to support the successful integration of nurses from diverse backgrounds into healthcare institutions who have achieved high levels of EI due to cultural differences.

This study is part of ongoing research on nursing students’ EI and self-esteem in Saudi Arabia and India. This study aimed to contribute useful information for designing curricula and support services that will be beneficial to future nurses. Moreover, cross-cultural comparison provides a broader basis for understanding how cultural background might affect EI and self-esteem, as well as indicating the relationships between these factors among selective demographic profiles. A deeper and more valid knowledge of the association between self-esteem, EI, and selected demographic variables can help educators or healthcare givers come up with effective methods of assisting culturally unique nursing students. This is crucial because currently, there are few investigations on the levels of emotional intelligence or confidence among Indian and Saudi Arabian nursing trainees who hail from diverse cultural backgrounds. However, gaps identified in the literature have limited empirical evidence necessary for improving nursing education while supporting learners, although it is widely recognized that more needs to be done in this area [7, 17]. Therefore, this investigation was conducted with the aim of appraising emotional intelligence (EI) together with self-esteem among Indian and Saudi Arabian undergraduate nurses towards enhance our understanding of their interrelations during nursing education/training program delivery. Consequently, this study sought to assess the levels of EI and self-esteem among Saudi Arabian and Indian nursing students to foster a more comprehensive understanding of their relationship during nursing education and practice.

## Methods

### Design

This study employed a descriptive–comparative research design to investigate the EI and self-esteem of nursing students in Saudi Arabia and India.

### Participants

Nursing students were recruited from universities A and B in Saudi Arabia and university C in India. Specifically, a convenience sample of 660 nursing students was recruited from September 2022 to January 2023. The participants had to satisfy the following inclusion criteria: willingness to participate, understanding English, studying both nursing theory and practice, and attending university for at least a year. Nursing students on leave during the study period were excluded.

### Data collection

The required data were gathered through an online survey comprising two self-reported questionnaires delivered via Google Forms. English was used in the online survey. An informed consent statement outlining the study’s purpose, methodology, risks, and benefits, as well as an agreement to participate, was included in the survey. Participants were encouraged to contact the researchers if they required clarification. The researchers provided a link to the survey to all nursing students who met the study’s inclusion criteria via Messenger and WhatsApp. Data collection was conducted from September 2022 to February 2023.

### Questionnaires

The first questionnaire used in this study was the Hall Emotional Intelligence Test (HEIT) [18], which comprises 30 statements answered on a scale ranging from completely disagree (–3 points) to completely agree (+ 3 points). For each statement, the sum of the points was calculated, considering the sign of the answer (+ or –). The greater the positive sum of the points, the more pronounced is the emotional manifestation. The statements were divided into four categories: emotional awareness (Items 1, 2, 4, 17, 19, and 25), managing emotions (Items 3, 7, 8, 10, 18, and 30), self-motivation (Items 5, 6, 13, 14, 16, and 22), and empathy (Items 9, 11, 20, 21, 23, and 28). The integrated indicator of EI, taking into account the dominant sign, was determined based on the following quantitative indicators: 70 or more = high, 40–69 = medium, and 39 or less = low. The validity and reliability of the questionnaire were also assessed. Here, three experts (two working as psychometricians in academia and one as an educational psychologist) validated the questionnaire and unanimously agreed that its contents were valid in assessing EI.

The Rosenberg Self-Esteem Scale (RSES) [19] was the second questionnaire used in this study. It is a 10-item self-report questionnaire that is widely used to measure individuals’ overall self-esteem. It is scored on a four-point Likert scale ranging from ‘strongly agree’ to ‘strongly disagree, with higher scores reflecting higher self-esteem. The item scores are summed to obtain the overall score, which can range from 0 to 30, with scores between 15 and 25 generally considered within the normal range.

We conducted a face validity assessment to ensure that the questionnaires captured the research questions on emotional intelligence (EI) and self-esteem. To measure these constructs, four specialists, psychometricians, and research professionals examined the tools and arrived at a consensus that the tools seemed appropriate. Moreover, a reliability testing was conducted prior to actual data collection with 20 student nurses in Saudi Arabia and 20 students in India with the Cronbach’s alpha of 0.88 and 0.83 respectively which indicates their high reliability.

### Data analysis

Data were analyzed using SPSS version 24. Moreover, an independent t-test and multivariate regression analysis were used to identify the associations between demographic factors and their relationships with the participants’ EI and self-esteem scores.

### Ethical considerations

Ethical approval for this study was obtained from all the three nursing schools. The participants were informed about the study’s objectives and processes, and they were informed that their participation was entirely voluntary and confidential. This study was approved by the Research Ethics Committee (ECM# 2021–4901) of King Khalid University, dated April 15,2021 and Apollo College of Nursing (H-01-R-059) dated March 8, 2021.

## Results

A total of 660 nursing students participated in this study. The participants were equally divided into second, third, and fourth years in Saudi Arabia and India. The majority of participants from Saudi Arabia and India were aged over 20 years old (56.5% and 56.8%,) respectively, were from urban residential areas (76.4% and 76.3%), and reported good academic performance (87.2% and 82%) (Table 1).

**Table 1:** Background Characteristics of Nursing Students. (*N* = 660)

Variables	Saudi Arabia (*n* = 375)		India (*n* = 285)	
	f	%	f	%
**Year of Study**				
Second year	125	33.3	95	33.3
Third year	125	33.3	95	33.3
Fourth year	125	33.3	95	33.3
**Age in Years**				
≤ 20	146	38.9	119	41.8
21–25	212	56.5	162	56.8
26–30	3	0.8	4	1.4
> 30	14	3.75	0	0
**Residence**				
Urban	282	76.4	214	76.3
Rural	93	23.6	71	23.7
**Academic Performance**				
≤ 50	2	0.5	0	0.0
51–59	0	0	0	0.0
60–74	46	12.3	71	24.9
≥ 75	327	87.2	214	82.0

Table 2 presents the differences between emotional intelligence, self-esteem, and background characteristics. There was a significant difference between emotional intelligence and year of study (*P* < .011), age in years (*p* < .024), residence (*p* < .005), and academic performance (*p* < .008). Regarding self-esteem, only age in years was found to have a significant difference (*p* < .002), while years of study (*p* > .670), residence (*p* > .430), and academic performance (*p* > .526) were not significantly different.

**Table 2:** Differences between emotional intelligence, self-esteem and background characteristics

Emotional intelligence					Self-esteem			
Variables	Mean	SD	F/ t value	*p* value	Mean	SD	F/t value	*p* value
**Year of Study**			F = 4.524	**0.011**			F = 0.401	0.670
Second year	60.58	21.12			27.60	3.93		
Third year	63.34	19.52			27.81	4.02		
Fourth year	66.47	18.96			27.47	4.27		
**Age in Years**			F = 3.160	**0.024**			F = 5.129	**0.002**
≤ 20	61.43	20.47			27.58	3.85		
21–25	64.31	19.52			27.47	3.97		
26–30	61.67	14.83			25.86	5.01		
> 30	72.60	19.30			30.33	5.86		
**Residence**								
Urban	64.77	19.45	t = 2.81	0.005	64.77	19.45	t=-0.79	0.430
Rural	59.53	21.15			59.53	21.15		
**Academic Performance**								
≤ 50	57.0000	27.01	F = 4.92	**0.008**	0.71	0.50	F = 0.642	0.526
51–59	0	0			0.00	0.00		
60–74	58.3063	21.70287			3.33	0.31		
≥ 75	64.7373	19.30211			4.22	0.18		

Table 3 presents the association between nursing students’ EI scores and the assessed demographic variables. More specifically, nursing students’ EI scores were significantly associated with the following variables: year of study, age, area of residence, and level of academic performance (*p* < .05). In other words, the EI scores were significantly higher among nursing students in more advanced years of study (years III and IV), aged over 20 years, from urban residential areas, and with good academic performance when compared with their peers.

**Table 3:** Correlation between nursing students’ EI and self-esteem scores

Variables	*r* Value	*p* Value
**EI vs. Self-Esteem**	0.265	0.000

Table 4 presents the results of the multivariate regression analysis of the nursing students’ EI and SE scores. Both the area of residence and academic performance were found to be significant predictors of EI (*p* < .05); that is, EI scores were found to be significantly higher among nursing students from urban residential areas who reported good academic performance (*p* < .05). Conversely, none of the assessed demographic variables were significant predictors of self-esteem (*p* > .05).

**Table 4:** Multivariate regression analysis of EI and SE scores among nursing students

Emotional Intelligence							
Variables	Unstandardized Coefficients		Standardized Coefficients	t	Sig.	95% CI for B	
	B	Std. Error	Beta			Upper Bound	Lower Bound
Country	-1.897	1.618	-.047	-1.172	0.242	-5.075	1.281
Year of Study	3.895	2.149	.090	1.813	0.070	-.324	8.115
Age	.832	2.065	.020	.403	0.687	-3.224	4.888
Area of Residence	-4.635	1.870	-.099	-2.479	0.013	-8.307	-.963
Academic Performance	4.918	2.126	.095	2.313	0.021	.743	9.094
Self Esteem							
Variables	Unstandardized Coefficients		Standardized Coefficients	t	Sig.	95% CI for B	
	B	Std. Error	Beta			Upper Bound	Lower Bound
Country	.434	.327	.053	1.326	0.185	-.209	1.076
Year of Study	-.016	.430	-.002	-.038	0.970	-.860	.827
Age	.018	.417	.002	.044	0.965	-.800	.837
Area of Residence	.348	.380	.036	.917	0.359	-.398	1.095
Academic Performance	.487	.433	.046	1.124	0.261	-.364	1.338

## Discussion

The present study sought to investigate the levels of EI and self-esteem among nursing students from two distinct cultural contexts: Saudi Arabia and India. The study found that EI levels varied among nursing students depending on their year of study, age, residence, and academic performance. According to these results, emotional intelligence can be developed in nursing students through maturity, life experience, the educational environment, and academic achievement. These findings align with a previous investigation undertaken by Budler et al. [7], who discovered that there tends to be an increase in emotional intelligence levels as years of study advance; this is also connected with other factors such as age, residence, or academic performance. Other studies have found that factors such as age, year of study, and life experiences can also contribute to the development of EI in nursing students over time [20]. This implies that exposing them to various clinical settings while challenging them academically will help them grow their EI skills over time. Moreover, it may be argued that with growth, people become more emotionally intelligent because they have seen many things happening around them; hence, they know how best to react. Moreover, urban areas provide better opportunities for personal development, which could affect one’s ability to develop certain types of EIs, depending on where they live. It has been observed too that Higher academic achievement is associated with greater self-awareness among those pursuing nursing courses [7]. For instance, if a student does well academically, it means that he/she possesses good problem-solving skills that contribute positively towards their general emotional intelligence. Therefore, education systems for nurses should create an atmosphere where they feel supported to nurture their awareness of emotions. Alternatively, training programs focused on teaching individuals to manage feelings effectively might be incorporated into curricula meant to foster emotional growth among learners [8]. In this context, enhancing EI and self-esteem among nursing students can lead to improved patient care, increased job satisfaction, and better retention of qualified nurses in the healthcare system.

Compared to their peers, nursing students in higher years — those older than 20 and coming from urban areas with strong academic records — tend to have greater emotional intelligence (EI) scores [7]. However, EI remains a popular research topic because of its broad impact on human life. The nursing community recognizes the importance of emotional intelligence for fostering communication skills, empathy, and quality patient care [7]. This indicates that nursing curricula should concentrate on factors affecting Emotional Intelligence in nursing students’ development [21]. In terms of educational planning, this calls for both nursing educators and policymakers alike to do the right thing [22]. Demographic and environmental factors can either positively or negatively predict EI development among student nurses, which is common knowledge. For instance, it was found that higher ages are associated with enhanced levels of Emotional Intelligence in seniority students, such as those aged between mid-twenties upwards, when compared to juniors like those in their early twenties [7]. This therefore implies that personal growth together with life exposure may affect EIs in them being nursing learners Another research indicated that urban settings have significant influence on EI formations among trainee nurses owing to their richness plus availability of resources needed for this kind education enhancement process according to different previous investigations various impacts were realized by academic levels towards emotional intelligence developments amongst healthcare undergraduates (e.g. being seniors scored higher than lower years [20]. This suggests that interventions tailored to foster self-awareness among these individuals must consider not only such variables but also others, such as age brackets and places where they come from, thus encouraging holistic growth within this sphere.

The present study found two variables— area of residence and academic achievement level—to be significantly associated with higher EI levels among nursing students. More precisely, nursing students who were urban residents had higher EI scores than their rural peers, while nursing students who reported better academic achievement also showed higher levels of EI than their peers. However, these findings do not directly suggest the cause-and-effect nature of the relationship between these factors and EI, although they do provide interesting insights concerning the potential influences on the development of EI. These findings confirm what has been found in other studies: EI is seen as an excellent forecast of mental and physical wellness across different populations [23]. In fact, high levels of emotional intelligence coupled with self-worth are deemed necessary for general welfare. People possessing such attributes usually tend to adopt healthier ways of living, which eventually results in improved overall physical health. In addition, these findings are consistent with research indicating that residents who have taken a gap year before starting medical school and women in certain residency programs have higher levels of EI [24], which indicates the possible influence of the environment and particular personal qualities on the development of EI, revealing the need for additional research in this area. Accordingly, emotional intelligence is a crucial factor in nursing students’ performance and retention, helping them overcome clinical obstacles, enhancing patient care, and developing effective communication and empathy skills [25]. Integrating EI and self-esteem-related content into the nursing curriculum, providing targeted training and mentorship programs, and addressing factors such as the urban-rural divide can help promote these essential attributes.

Emotional intelligence among nursing students is significantly affected by the educational environment and academic performance. Moreover, higher EI scores were found in those students from urban areas who reported good academic performances which imply that these two factors can predict for someone’s EI [4]. Based on this finding, it is important to take care about caring environments for education and diverse support systems since they contribute greatly in building self-esteem among the nursing students’ themselves thus promoting their general wellbeing. The relationship between EI and nursing student’s self-esteem is very essential as this will greatly influence how well they perform their duties when they become nurses later in life. For instance, good patient care requires emotional intelligence which includes better communication skills such as being able to empathize with others’ feelings or resolving conflicts amicably (i.e. listening to understand rather than just respond) since these are some of the things that can make one succeed or fail when dealing with patients suffering from different kinds of illnesses or diseases. People who possess high levels of EI tend to have strong critical thinking abilities during learning process therefore individuals who are confident about themselves also tend to think more critically because they know how best manage their emotions during difficult times [26], learners who take an active part in school work usually do well academically hence staying positive towards education even after realizing that it did not help them much in terms of career development [4]. This study has great implications for practice concerning emotional intelligence (EI) and self-esteem among nursing students. For example, such programs should be supported where emotional intelligence can be nurtured along with peer feedback through mentorship programs that foster self-esteem within healthcare institutions as highlighted by this research paper. Intervention strategies aimed at enhancing levels of emotional intelligence among urban based nursing learners include improving upon academic performance; thus there should be setting up tutoring units or increasing teachers teaching such subjects (i.e. English language). The findings further indicate that age, gender, and socio-economic status have little impact on nurses’ personal worth. This finding is consistent with previous studies that concluded that there is no significant relationship between demographic factors (age, sex, socioeconomic status, etc.) and self-esteem levels or dimensions [4]. This implies that the educational environment must always remain a critical factor in shaping emotionally intelligent individuals who are confident enough about themselves and are therefore capable of delivering quality services within any healthcare setting.

### Study implications

This study has important implications in improving EI through nursing education. Mindful activities and training should be added to nursing curricula and programs aimed at helping students develop their EI. Programmatic interventions target specific groups of learners who have self-esteem problems as well as those with emotional intelligence difficulties to create supportive systems within them while in college or school. The findings clearly indicate that countryside dwellers, second-year students in colleges/universities/other tertiary institutions, and those performing poorly academically require more frequent resources on emotional intelligence. Lecturers in the nursing profession can enhance cultural sensitivity through their teachings in addition to stimulating debates about self-worthiness among different ethnic communities or social clusters, given the diversity among these groups. Such conversations foster inclusiveness in an educational setting. Although this investigation had limitations regarding the countries involved or the cultures represented, its discovery could be repeated elsewhere worldwide. It would be interesting to examine what factors drive emotional intelligence and self-esteem among all categories of nursing scholars from various parts after comparing less developed and more developed/industrialized nations around the world.

### Study limitations

This study had several limitations. For example, the data may have selection biases, such as only students from specific universities in a convenient sample being included, which might not represent all nursing students in those countries. Moreover, the researchers took samples from India and Saudi Arabia; hence, it is uncertain whether these findings can be applied universally. Additionally, this research cannot be generalized because of differences in the way nurses interpret emotional intelligence (EI) and self-esteem across different cultures worldwide. In line with this, there are great differences between various parts of India, including their languages and dressing codes, but we still share some common values, especially when it comes to caring for patients’ health; hence, cultural exchanges among healthcare professionals should not only focus on one or two nations alone. In addition, undergraduate students were used as our sample because they are easier to find than postgraduate students who may be working already, and thus difficult to hold off for research purposes. Furthermore, while accessibility played a role, we prioritized recruiting from institutions with strong nursing programs to ensure a participant pool with a solid foundation in both theory and practice, which strengthens the generalizability of our findings within the context of undergraduate nursing education in these countries where most of such schools exist. However, future studies need to extend their scope by including other regions within India as well outside its borders, particularly areas with different program structures or student demographics alongside Saudi Arabia’s diverse cities.

## Conclusion

Higher emotional intelligence among nursing students can be associated with several variables, such as being in the higher years of study, older age, residing in an urban area, and good academic performance, whereas self-esteem appears to be hardly affected by these indicators but probably influenced by other aspects that were not measured. This implies that educators in relation to self-esteem should know the relationship between emotional intelligence and nursing practice besides healthcare establishments; they need to enhance their teaching methods so that learners can have more resilient attitudes towards work, provide quality patient care, and promote a better learning environment for nurses who will become stronger professionally in the future.

## Data Availability

The datasets generated in this current study are available from the corresponding author on reasonable request.

## Abbreviations

EI: Emotional intelligence
HEIT: Hall emotional intelligence test
RSES: Rosenberg self-esteem scale
SE: Self-esteem
